# Access to diabetes diagnosis in Brazil based on recent testing and consultation: The Brazilian national health survey, 2013 and 2019

**DOI:** 10.3389/fendo.2023.1122164

**Published:** 2023-03-22

**Authors:** Karine Brito Matos Santos, Rodrigo Citton P. dos Reis, Bruce B. Duncan, Otávio Pereira D’Avila, Maria Inês Schmidt

**Affiliations:** ^1^ Postgraduate Program in Epidemiology, Universidade Federal do Rio Grande do Sul, Porto Alegre, Brazil; ^2^ School of Medicine, Universidade Estadual do Sudoeste da Bahia, Vitória da Conquista, Brazil; ^3^ Statistics Department, Universidade Federal do Rio Grande do Sul, Porto Alegre, Brazil; ^4^ Social Medicine Department, Universidade Federal do Rio Grande do Sul, Porto Alegre, Brazil; ^5^ Hospital de Clínicas de Porto Alegre, Porto Alegre, Brazil; ^6^ Postgraduate Program in Odontology, Universidade Federal de Pelotas, Pelotas, Brazil

**Keywords:** diabetes mellitus, diagnosis, health care, health inequities, cross-sectional studies

## Abstract

**Background:**

Screening for undiagnosed diabetes using glucose testing is recommended globally to allow preventive action among those detected. Our aim was to evaluate the access to glucose testing to screen for diabetes in Brazil using self-reported information on recent testing and medical consultation from national surveys of Brazilian adults.

**Methods:**

The *Pesquisa Nacional de Saúde (*PNS) was conducted in 2013 and 2019 drawing probabilistic samples of Brazilians aged 18 years and above. To evaluate glucose testing among those undiagnosed, we excluded those self-reporting a previous diagnosis of diabetes. We then defined recent access to diabetes diagnosis by considering the previous two years and choosing the last blood glucose test and the proximal medical consultation reported. We used Poisson regression with robust variance to assess correlates of access, expressing them with adjusted prevalence ratios (PR) and their 95% confidence intervals.

**Results:**

Access to recent glucose testing documented that over 70% reported a recent glycemic test, 71% in 2013, and 77% in 2019. These findings are consistent with a wide recent access to medical consultation, 86% and 89% in 2013 and 2019, respectively. Reporting recent glucose testing and medical consultation may better reflect the actual access to medical diagnostic testing. When analyzing this joint outcome, diagnostic access was still wide, 67% and 74%, respectively. Greater access (p< 0.001) was seen for women (PR=1.16; 1.15-1.17), older individuals (PR=1.25; 1.22-1.28), and those with higher education (PR=1.17; 1.15-1.18), obesity (PR=1.06; 1.05-1.08), and hypertension (PR=1.12; 1.11-1.13). In contrast, lower access (p<0.001) was seen for those declaring being Black (PR=0.97; 0.95-0.99) or of mixed-race (PR=0.97; 0.96-0.98), those residing in rural areas (PR=0.89; 0.87-0.90), and not having a private health insurance plan (PR=0.85; 0.84-0.86).

**Conclusions:**

Although access to diagnostic testing for diabetes is high in Brazil, partly due to its universal health system, social inequities are still present, demanding specific actions, particularly in rural areas and among those self-declaring as being Black or mixed-race.

## 1 Introduction

Diabetes is a chronic disease with a global impact. By 2021, 537 million people worldwide had diabetes. The growth in cases is skyrocketing, with an estimated 783 million people having diabetes by 2045. The projected increase in cases appears to be due to projected population aging and growth, urbanization, lifestyle, and environmental pollution, among other factors (1). Diabetes also is responsible for a great burden, placing diabetes among the principal causes of loss of health. For instance, in 2019, in the Americas, it was estimated that 409,000 adults aged 20 years or older died from diabetes (5-9% of all deaths). Diabetes was responsible for 2266 crude disability-adjusted life-year (DALYs) per 100,000 adults in the Americas (2). Owing to the frequently long period between the onset of the disease and the onset of diabetes symptoms, a considerable proportion of type 2 diabetes cases remain undiagnosed, leading to increased mortality, diabetes-related complications, and costs (1). Behavioral risk factors such as low physical activity and unhealthy diets are the main determinants of diabetes and its complications (3–5).

Not having consulted a doctor in the last year is one of the main determinants of the delayed diagnosis of mild and asymptomatic cases of diabetes (6). Therefore, the American Diabetes Association (ADA) recommends tracking diabetes in all individuals over the age of 35 or at any age for overweight/obese adults who have at least one additional risk factor for diabetes. Screening can be done directly by asking for a glycemic test for all, or in two steps, by applying the glycemic test only to those at higher risk by questionnaire. The ADA also recommends repeating the test every three years or more often for those at high risk (7). The Brazilian Society of Diabetes follows similar criteria, except for the age of screening, using a threshold of 45, instead of 35 years old (5).

In order to effectively act in the health-to-disease course, adequate access to health services is essential (8). Access is usually defined by the timely use of health services to achieve the best possible health outcomes (9). On the premise that health is a right of all citizens, ensuring universal access to cost-effective health services is mandatory and thus requires regular evaluation. To our knowledge, assessment of access to diabetes diagnosis has been assessed using nationwide representative samples in the United States and Puerto Rico, Argentina, and Sub-Saharan countries, with rates ranging from 77% to 22% (in decreasing order) (10–12).

To gain insight into the population coverage of glucose testing for the diagnosis of diabetes, our objective is to evaluate the access to glucose testing and medical consultation in Brazil in 2013 and 2019 using self-reported information on recent testing and medical consultation available in the *Pesquisa Nacional de Saúde (*PNS), a household national representative survey of Brazilian adults. In addition, we aimed to relate access to demographic, socioeconomic, and clinical factors.

## 2 Methods

### 2.1 The PNS surveys

The PNS is a national population-based household survey conducted by the Brazilian Institute of Geography and Statistics (IBGE) in partnership with the Ministry of Health, which has been conducted twice, in 2013 and 2019. The selection of participants was based on cluster probability sampling in three stages of selection and stratification of the primary sampling units (PSUs). The PSUs are formed by census tracts or composition of census tracts; the second stage units being households, selected to produce a fixed number of permanent private households for each PSU; the third stage units are residents aged 18 years or older (2013) and 15 years or older (2019), selected from a list of residents built at the time of the interview. For each one of the three stages, a simple random sampling was performed for the selection of the units. More information about the design of the surveys can be found elsewhere (13, 14).

Because of its complex sample design, and to estimate population parameters, the expansion factors or basic sample weights for the households, all residents, and the selected resident were provided for the PNS Surveys by IBGE. The basic weights were adjusted to correct for non-response, and calibrated according to Brazilian population projections by gender and age group (14, 15). In order to dimension the sample size with the desired level of precision for the 2019 PNS estimates, the IBGE considered some indicators of the 2013 edition of the PNS, such as data on non-communicable chronic diseases (NCDs) (diabetes, hypertension, depression), violence, use of health services, possession of health insurance, smoking, physical activity practice, and alcohol consumption, among others (14). The microdata files are available from the PNS website (16). For this study, we used data for adults 18 years or older.

### 2.2 Analytic sample

**Figure 1:**
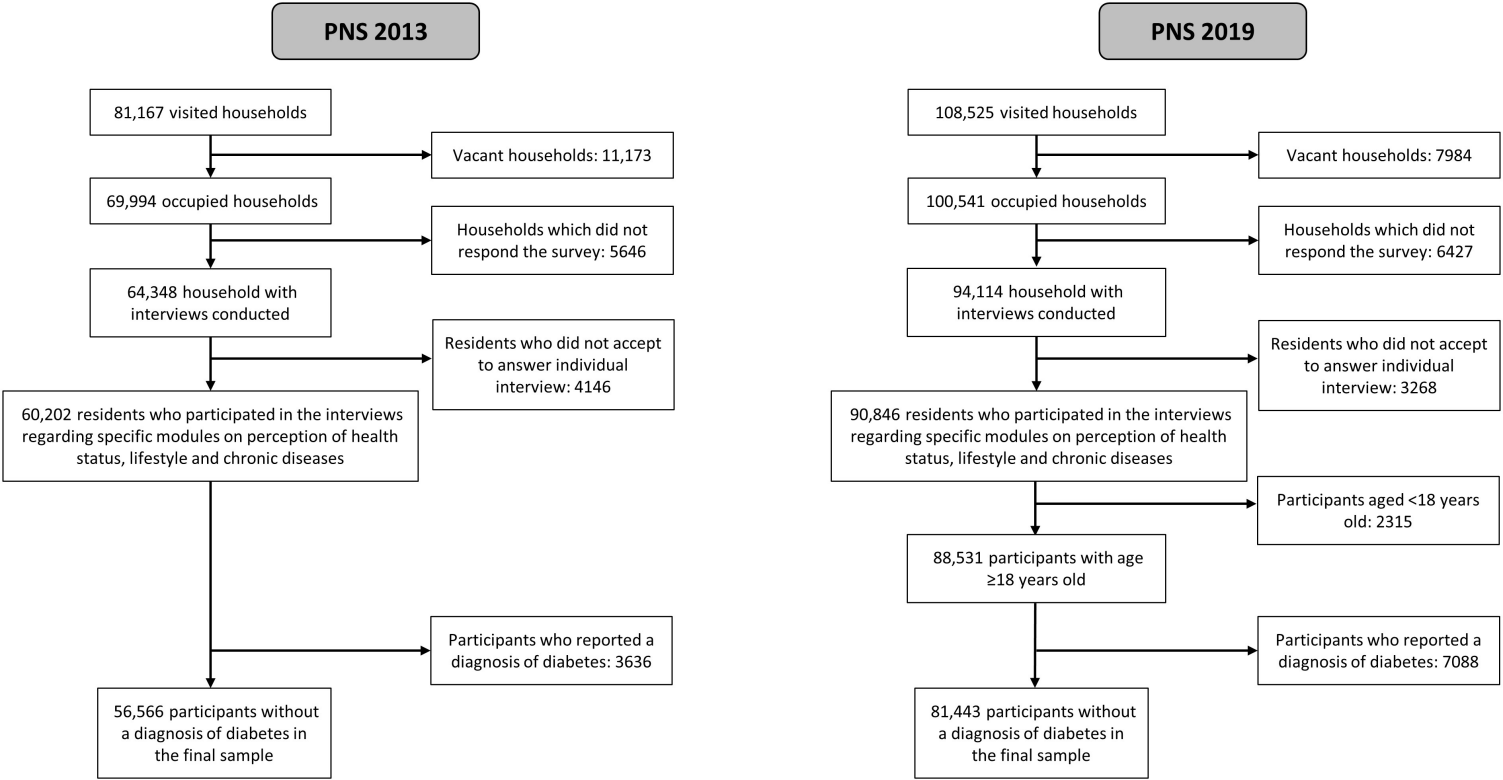
Flowchart of study participants, Brazilian National Health Survey, 2013 and 2019.

**Figure 1** shows the sample flowchart of the Brazilian National Health Surveys conducted in 2013 and 2019. In 2013, of the 81,167 households selected, 11,173 were empty, 5646 did not answer the survey and 4146 individuals did not agree to answer the individual questionnaire, leaving 60,202 residents aged 18 years or older who answered the individual questionnaire. This corresponds to a response rate of 86% of total non-empty selected households. Of these, 3636 (6.03%) reported having diabetes, and 56,566 not having this diagnosis. In 2019, of the 108,525 households selected, 7984 were empty, 6427 did not answer the survey, and 3268 did not agree to answer the individual questionnaire, leaving 90,846 residents aged 15 years or older as respondents (~90% of total non-empty selected households). For this study we included respondents aged 18 years or older, which corresponded to 88,531 residents; of these, 7088 (6.53%) people reported having a diagnosis of diabetes, and 81,443 not having.

### 2.3 Measurements

The PNS questionnaire was divided into 20 modules in 2013, and 26 modules in 2019, and included characteristics of the households, all residents, and the selected resident. We used the following questions to analyze aspects describing access to glucose testing and medical consultation for those not reporting a previous diagnosis of diabetes. “When was the last time that you had a blood test to measure blood glucose, that is, blood sugar?” (Questions Q29 and Q29a, in 2013 and 2019, respectively); and “When was the last time that you consulted with a doctor?” (Questions J11 and J11a, in 2013 and 2019, respectively). For the last blood glucose test, the response options were as follows: less than 6 months, between 6 months and 1 year, between 1 and 2 years, between 2 and 3 years, 3 years or more, and never performed. For the medical consultation, the answer options were as follows: in the last 12 months, between 1 and 2 years, between 2 and 3 years, 3 years or more, and never performed.

We defined access to glucose testing (yes or no) for the detection of diabetes among those not previously diagnosed by a report of a glucose test within two years of the interview. Since screening for diabetes is recommended to occur every 1-3 years, we judged that a two-year period could be recent enough to characterize adequate screening. To evaluate the robustness of this glucose testing assessment to define access to medical diagnosis we also evaluated the joint occurrence of a recent glucose testing and a recent medical consultation.

In the 2013 edition, weight and height were measured, while in 2019, these variables were self-reported. Demographic, socioeconomic, and clinical factors were also obtained from the PNS questionnaires. Sociodemographic characteristics - sex: male and female; age group in years: 18-24, 25-39, 40-59, and 60 or greater (≥60); race/color: white, black, brown (mixed-race), Asian (yellow), and indigenous; education: with no formal education or incomplete elementary school (incomplete elementary), complete elementary school or incomplete high school (complete elementary), complete high school or incomplete higher education (complete high school), and complete higher education; geographic macro-region: Central-West, Northeast, North, Southeast, and South; type of census situation: urban and rural; having private health insurance: yes and no. Clinical factors - body mass index (BMI), calculated as weight in kilograms divided by height in meters squared: Low Weight/Normal (< 25 kg/m^2^), Overweight (between 25 and 29.9 kg/m^2^), and Obesity (≥ 30 kg/m^2^); the presence of hypertension: yes and no; diagnosed diabetes: yes and no.

### 2.4 Statistical analyses

To compare the results of the 2013 and 2019 PNS surveys, the IBGE recalibrated the PNS 2013 sample weights, based on the revised Brazilian population projection by gender and age used for the 2019 survey (17). The data of the two PNS editions were combined, using the survey year as a covariate, and making adjustments in the sample weights as suggested by Korn and Graubard (1999) (18), similar to those adopted in other studies (19, 20).

Sociodemographic and clinical characteristics were described by simple frequencies and percentages weighted by calibrated weights, provided together with the datasets by IBGE. The distribution of access variables to diagnostic services was described by weighted percentage and 95% confidence interval (95% CI). Comparisons of sociodemographic and clinical characteristics between the two editions of the PNS survey were evaluated using a chi-square test with the Rao-Scott adjustment (21).

The associations of access outcomes with sociodemographic and clinical characteristics were evaluated using adjusted prevalence ratios (PR) and 95% CI, estimated by Poisson regression models with robust variance. We built progressively larger models by adding factors likely to be related to access in the following order: gender, age, race/color, education, geographic macro-region, type of census situation, having private health insurance, and clinical conditions such as levels of BMI and hypertension. We checked for possible collinearity across the independent variables using the generalized variance inflation factor (GVIF) (22). We considered a threshold of 2.5 (VIF >2.5) as indicative of the need for further evaluation (23).

Data analysis was performed in the statistical software R (24), version 4.0.4 with the survey package (25) to take into account the complex sample design.

## 3 Results

We found a slight predominance of women; most were between ages 25 to 59 and declared to be White or mixed-race (**Table 1**). Few had completed higher education (13% and 16.4% in 2013 and 2019, respectively). Between 2013 and 2019 we observed an increased frequency of people with age 60 years old or over and with completed high school. We also noticed a slight increase in overweight and obesity, as well as a slight increase in a self-reported diagnosis of hypertension between surveys. About a third of the population had private health insurance in both survey years.

**Table 1:** Sociodemographic and clinical characteristics of participants, without diabetes diagnosis [n (weighted %)], in the Brazilian National Health Survey, 2013 and 2019 (n = 138,009).

Characteristics	2013 n (%)	2019 n (%)	*p*-value^†^
Overall	n = 56,566	n = 81,443	-
Sex			0.549
Male	24,639 (47.6)	38,784 (47.3)	
Female	31,927 (52.4)	42,659 (52.7)	
Age (years)			<0.001
18-24	7789 (16.9)	8090 (14.9)	
25-39	20,486 (33.5)	25,072 (31.2)	
40-59	19,010 (33.9)	29,827 (35.2)	
≥60	9281 (15.8)	18,454 (18.7)	
Race/Color*			<0.001
White	22,550 (47.3)	29,675 (43.1)	
Black	5216 (9.0)	9253 (11.5)	
Mixed-race	27,904 (42.3)	41,292 (44.0)	
Yellow	504 (0.9)	597 (0.9)	
Indigenous	390 (0.4)	617 (0.5)	
Education			<0.001
Incomplete elementary	21,858 (37.6)	31,323 (32.8)	
Complete elementary	8774 (15.7)	11,226 (14.7)	
Complete high school	18,511 (33.8)	25,987 (36.1)	
Complete higher education	7423 (13.0)	12,907 (16.4)	
Region			0.321
Central-West	7027 (7.4)	9353 (7.6)	
Northeast	17,236 (26.7)	28,286 (26.6)	
North	11,998 (7.6)	15,895 (8.0)	
Southeast	13,244 (43.5)	17,603 (43.1)	
South	7061 (14.8)	10,306 (14.7)	
Census situation			0.958
Urban	46,152 (86.0)	62,484 (86.0)	
Rural	10,414 (14.0)	18,959 (14.0)	
Private health insurance			0.607
Yes	15,287 (30.0)	20,510 (29.7)	
No	41,279 (70.0)	60,933 (70.3)	
Body mass index*			0.209
Low Weight/Normal (< 25 kg/m^2^)	24,510 (44.3)	34,842 (43.3)	
Overweight (between 25 and 29.9 kg/m^2^)	20,215 (36.0)	29,965 (36.5)	
Obesity (≥ 30 kg/m^2^)	11,049 (19.7)	15,795 (20.1)	
Hypertension			<0.001
Yes	10,252 (18.6)	17,959 (20.6)	
No	46,314 (81.4)	63,484 (79.4)	

*n slightly smaller due to missing values: Race/Color (n_missing_ = 11); Body mass index (n_missing_ = 1633).

^†^Rao-Scott chi-square test.

The descriptive data presented in **Table 2** show ample access to blood glucose testing over the two years prior to the interview (2013 and 2019) among those not reporting a previous diabetes diagnosis, with a slight increase in the last survey (71.1% to 77.2%). The percentage of those who reported never having done a glucose test was small in 2013 (12.3%) and decreased to 6.8% in 2019. These data are consistent with a broad report of medical consultation in the 2 years before the study (85.6% and 89.2%, respectively). The percentage of that who reported never having had a medical consultation was minimal (0.8% and 0.6%, respectively).

**Table 2:** Weighted percentage (95% CI) of adults without diabetes diagnosis, according to the time since the last medical consultation and last blood glucose test, in the Brazilian National Health Survey, 2013 and 2019 (n = 138,009).

Survey question	2013 % (95% CI)	2019 % (95% CI)	*p*-value^†^
Overall	n = 56,566	n = 81,443	-
When was the last time you had a blood test to measure your blood glucose?			<0.001
Less than 2 years	**71.1 (70.3, 71.9)**	**77.2 (76.6, 77.7)**	
2 years or more	16.6 (16.0, 17.1)	16.1 (15.6, 16.5)	
Never did	12.3 (11.8, 12.9)	6.8 (6.4, 7.1)	
When was the last time you saw a doctor?			<0.001
Less than 2 years	**85.6 (85.1, 86.2)**	**89.2 (88.8, 89.6)**	
2 years or more	13.6 (13.0, 14.1)	10.2 (9.8, 10.6)	
Never had been with a doctor	0.8 (0.6, 0.9)	0.6 (0.5, 0.7)	
Most recent consultation/blood glucose test			<0.001
< 2 years/< 2 years	**66.8 (66.0, 67.6)**	**73.8 (73.1, 74.4)**	
2 years or more/< 2 years	4.1 (3.9, 4.4)	3.3 (3.0, 3.5)	
Never been to doctor/< 2 years	0.2 (0.1, 0.2)	0.2 (0.1, 0.2)	
< 2 years/2 years or more	10.6 (10.1, 11.0)	10.8 (10.4, 11.2)	
2 years or older/2 years or more	5.8 (5.5, 6.2)	5.1 (4.8, 5.4)	
Never been to doctor/2 years or more	0.2 (0.1, 0.2)	0.2 (0.1, 0.2)	
< 2 years/Never did	8.3 (7.8, 8.7)	4.6 (4.4, 4.9)	
2 years or more/Never did	3.6 (3.3, 3.9)	1.8 (1.7, 2.0)	
Never been to the doctor/Never did	0.4 (0.3, 0.5)	0.3 (0.3, 0.4)	

^†^Rao-Scott chi-square test. The prevalence of outcomes (i.e., Recent blood glucose test, Recent medical consultation, and Recent glucose and consultation) are highlighted in bold.

To complement our assessment of the access to glucose testing as a mean of screening for diabetes we considered the joint occurrence of a recent glucose test and a recent medical consultation. Although frequencies were lower than when assessing only the frequency of glucose testing, they remained high (67% and 74%, each year, respectively).

**Figure 2:**
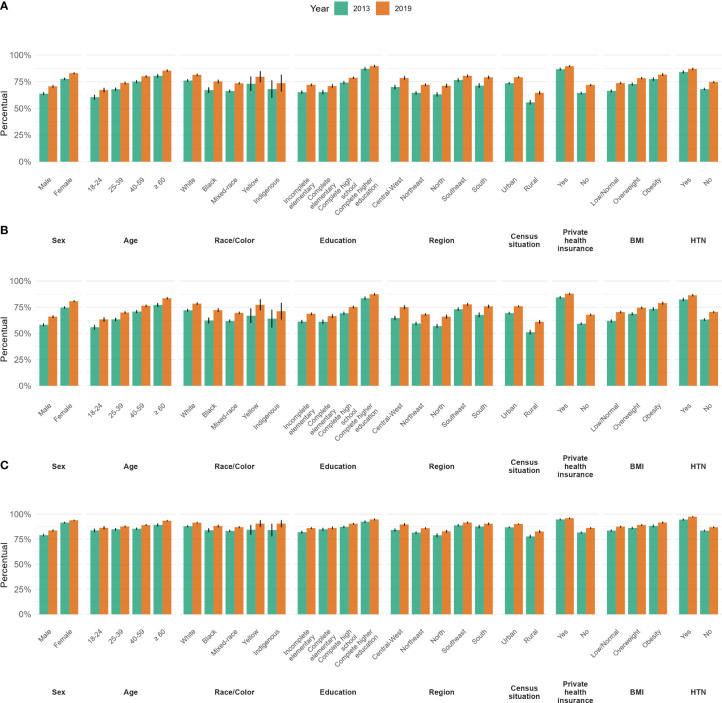
Frequency of recent (over the previous 2 years) access to a diagnosis of diabetes by sociodemographic and clinical characteristics considering three options to define access: **(A)** Glucose test, **(B)** Glucose and medical consultation, **(C)** Medical consultation, Brazilian National Health Survey, 2013 and 2019. BMI, Body mass index; HTN, Hypertension.

**Figure 2** illustrates the wide access to diabetes diagnosis for three measurements of access: (A) Last glucose test <2 years; (B) Last glucose test <2 years and Last consultation <2 years; (C) Last consultation <2 years, according to various characteristics. Access was generally higher in women, those aged 60 years or older, those with higher education, living in urban areas, and having private health insurance.

**Figure 3:**
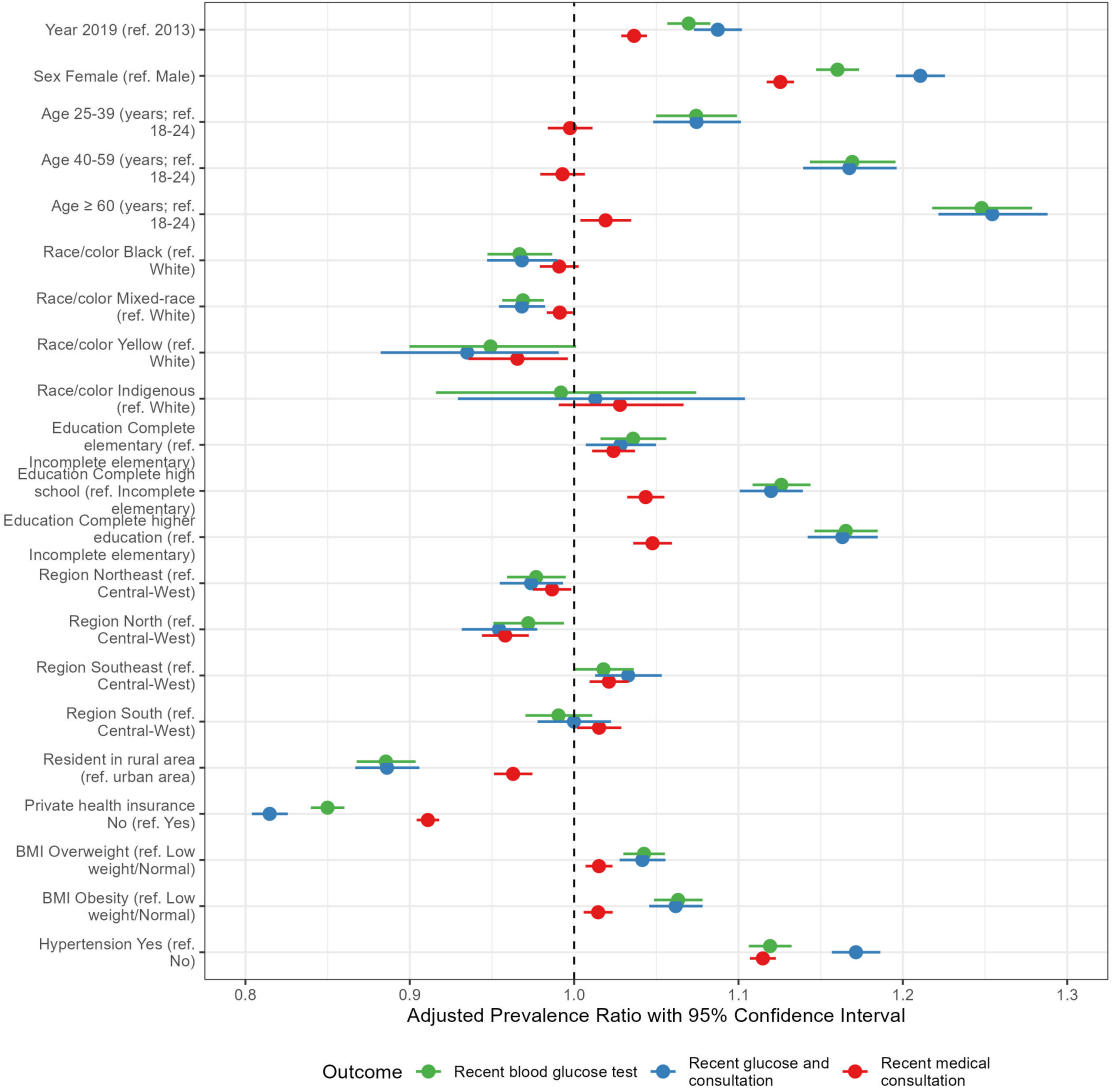
Association of survey year and sociodemographic and clinical characteristics with access to a recent glucose test, a recent medical consultation, or both, adjusted for gender, age, education, race/color, geographic macro-region, living in an urban or rural area, having private health insurance, and the year of the survey, as well as BMI and hypertension; the dashed vertical line represents PR = 1.0, meaning no association; PR values on the right side of the dashed line mean factors increasing the prevalence of recent access; otherwise, PR values on the left side of the dashed line mean factors associated with a lower prevalence of recent access. Brazilian National Health Survey, 2013 and 2019. BMI, Body mass index.

As illustrated in **Figure 3**, and described in detail in **Supplementary Table 1**, access to a recent glucose test was relatively higher in 2019 (PR=1.07; 1.06-1.08), consistent with the increase in recent consultation (PR=1.04; 1.03-1.04). Access to a recent glucose test was higher in women (PR=1.16; 1.15-1.17), those 60 years or older (PR=1.25; 1.22-1.28), with complete higher education (PR=1.17; 1.15-1.18), obesity (PR=1.06; 1.05-1.08) and a previous diagnosis of hypertension (PR=1.12; 1.11-1.13). Access to a recent glucose test was lower in people of Black (PR=0.97; 0.95-0.99) or mixed-race (PR=0.97; 0.96-0.98), living in rural areas (PR=0.89; 0.87-0.90) and without private health insurance (PR=0.85; 0.84-0.86). The report of a recent medical consultation showed a similar pattern of association.

When the recency of glucose testing was considered together with a reporting of a recent consultation, the associations showed a similar pattern. Access to a recent blood glucose test was relatively higher in 2019 (PR=1.09; 1.07-1.10), women (PR=1.21; 1.20-1.23), people 60 years of age or over (PR=1.25; 1.22-1.29), with a complete higher education (PR=1.16; 1.14-1.18), with obesity (PR=1.06; 1.05-1.08) and with diagnosed hypertension (PR=1.17; 1.16-1.19). It was relatively lower in people of yellow (Asian) race/color (PR=0.93; 0.88-0.99), living in rural areas (PR=0.89; 0.87-0.91), without a private health insurance plan (PR=0.81; 0.80-0.83) and living in the North region (PR=0.95; 0.93-0.98).

## 4 Discussion

Our findings from the PNS 2013 and 2019 show generally wide access to screening and diagnosis of diabetes in Brazil. Access is greater in women, the elderly, those living in the Southeast region, and those with overweight, obesity, and hypertension. These findings reflect the wide access to medical consultation in the two years before the interview. However, inequities in access related to low education, self-declaring as being Black, and living in rural areas and the North region warrant further attention.

The Unified Health System (*Sistema Único de Saúde* – SUS), implemented after the new constitution of 1988 in Brazil, provides universal access to all levels of health care, with a broad coverage of primary health care, the preferred gateway to health care in SUS. Between 2013 and 2019, coverage increased by 6.5 percentage points, from 56.1% to 62.6%, which corresponds to a proportional increase of 11.6%, with the inclusion of an additional 18.7 million residents in the Family Health Strategy (26). This may explain the ample access to medical consultation reported and the consequent large access to glucose testing described here.

For comparison, another Brazilian survey, Vigitel, conducted in state capitals in 2011 found a similar rate of recent glucose testing (76%). Factors related to higher testing were also similar (27). Of note, however, Vigitel data refer to those living in capital cities and thus its finding reflects more our specific results for urban areas (in 2013, 73.6%).

In the United States, a similarly high rate of recent (3 years) glucose testing was reported, 63.8% (11). In Argentina, as well, high rates were found (65.2% in 2009) (10). In sub-Saharan Africa, a pooled data analysis derived from nationwide samples found lower testing rates (only 22% of those overweight or obese had ever had glucose testing), being higher in countries with higher per capita income (12). Screening for undiagnosed diabetes based on glucose testing inevitably also detects prediabetes and this latter diagnosis may lead to overdiagnosis as well as unnecessary medical interventions (28, 29). A study developed in India revealed that HbA1C levels increase with age and points to the need to define age-specific cutoff points to avoid the risk of overdiagnosis and unnecessary initiation of treatment (30).

That greater access to diagnosis occurred in women, older people, and those with higher education is consistent with data from other studies (31–33). Perception of health needs has been shown to be an important indicator of access and use of health services (31, 34, 35) and may explain our findings. Women may have a greater perception of the importance of medical care, greater utilization of health services for monitoring prenatal care and the follow-up of children (31, 36, 37), and perhaps greater motivation to do check-ups and participate in health promotion and disease prevention activities. Those in a higher age group are likely to have another diagnosis of chronic disease demanding longitudinal care thus facilitating opportunistic testing. In contrast, younger people do not perceive themselves as at risk of developing some disease and seek fewer health services, and also have fewer symptomatic illnesses leading to consults. The expansion of the public network in Brazil occurred mainly for primary health care (PHC), expanding access to medical consultations for a substantial portion of the Brazilian population (38, 39). However, differences remain in the use of services that benefit those who have health insurance. Although our data show greater access to diagnosis in those with private health insurance, the difference between these two groups has been decreasing. In 1998, people with private health insurance plans were 200% more likely to use a health service when they perceived a need for it than people without health insurance, but this difference was reduced to 70% in 2008 (38). The new funding model of PHC, implemented in 2019, through weighted capitation and payment for performance, induces a more adequate identification of people linked to each family health team and imposes the improvement of indicators seeking better results in care, which allows us to envision the expansion of access in PHC (40). The population with health insurance plans may also have a greater opportunity to access services because many use both SUS and supplementary health services (31, 41).

Although the findings demonstrate broad diagnostic access in the country, some gaps observed deserve discussion. First, a percentage of people without a previous diagnosis of diabetes reported not having had a recent blood glucose test (<2 years), even though they had a recent medical consultation (<2 years), 14.5% in 2013 and 12% in 2019, with a proportional reduction of 17% in the period. Although this may represent a loss of diagnostic opportunity, periodic blood glucose application every 1-3 years, recommended in guidelines (7), can mitigate this gap. The SUS has been expanding access to health care (38), and the increase in the frequency of consultations is associated with increased diagnosis (42–45), which explains, at least in part, the reduction of diagnostic loss in the period. Second, our data also show gaps in access to diabetes diagnosis, especially sociodemographic factors, such as those living in rural areas, declaring themselves Black or mixed-race, or having low education. These inequities can be attributed in part to differences in behaviour when seeking health care. Groups with lower income and/or lower education may delay the decision to seek health care due to negative experiences obtaining care in the past or related to the care they received, or due to other factors, such as the impossibility of missing work or the perception of no need for health counselling (38). Often also, other priorities in their lives may take on greater importance. Interventions focusing on risk factors, added to actions in social determinants are necessary to expand access to diabetes diagnosis.

Our study has potential limitations. The first one refers to the cross-sectional design of the PNS survey that includes different participants in each sample, limiting the inferences of the associations that we report to the changes that occurred in the individuals studied. Second, data collection was based on self-reported information, and thus subject to information bias including recall bias. Although BMI calculations for the 2013 survey were based on measured weight and height, in 2019 they were based on self-report. Thus, misclassification may affect the associations here reported between the two years. Of note, however, a high agreement between self-reported and measured weight, height, and body mass index was observed in the PNS 2013 (46).

Important strengths of our analysis also deserve mention. The main one is the representativeness of our data, which allows the generalization of our findings for the Brazilian adult population. The large sample size of the research in the two years allows accurate estimates at the national level, as well as estimates, although less accurate, for other subgroups of the population. That two national health surveys have already been conducted makes it possible to evaluate the growing trend in access to health services for diagnosis and primary diabetes care.

Despite the limitations presented, this study contributes to otherwise sparse data on access to diabetes diagnosis, enabling debate on various dimensions of access to health services and their inequities, pointing to groups with greater barriers to the early detection of diabetes. Access and quality are inseparable in improving care for many health conditions, such as diabetes, being essential indicators in diagnosis and follow-up. The high percentages of diagnostic access to diabetes in the Brazilian population here described were possible, in large part, by the universal access to health care provided by the SUS. The SUS principles of universal access, comprehensiveness, and equity aim to guarantee the use and access of services by the whole population, thus including those with lower education and income, and without health insurance plans. However, as an underfinanced and developing health system, the SUS continues to struggle to ensure universal and equitable coverage, and there is much room for improvement.

In conclusion, access to screening and diagnosis of diabetes is high in Brazil, reflecting the wide access to medical consultation provided by the universal health system. However, inequities are still present, indicating the need for specific actions for specific groups, especially in rural areas and for Blacks and mixed-race groups.

## Data availability statement

Publicly available datasets were analyzed in this study. This data can be found here: PNS/Fiocruz website (https://www.pns.icict.fiocruz.br/bases-de-dados/).

## Ethics statement

Ethical review and approval was not required for the study on human participants in accordance with the local legislation and institutional requirements. Written informed consent for participation was not required for this study in accordance with the national legislation and the institutional requirements.

## Author contributions

KS and RR contributed to the study design, data analysis, interpretation of results, and writing the manuscript. MS contributed to the study design, interpretation of results, and writing the manuscript. BD and OD’A contributed to the data interpretation and reviewing of the manuscript for intellectual content. All authors contributed to the article and approved the submitted version.
