# Supplementary material for: Access to diabetes diagnosis in Brazil based on recent testing and consultation: The Brazilian national health survey, 2013 and 2019
Source: Front Endocrinol (Lausanne). 2023 Mar 22;14:1122164. doi: 10.3389/fendo.2023.1122164 (PMC10073740; doi:10.3389/fendo.2023.1122164)
Supplement: Supplementary file 1 [file Table_1.docx]

Supplementary Material

# Supplementary Tables

**Supplementary Table 1.** Adjusted association* of sociodemographic and clinical characteristics with a recent (<2 years) glucose test, medical consultation, or both in adults (≥18 years) without a previous diabetes diagnosis. Brazilian National Health Survey, 2013 and 2019 (n = 136,365).

| **Variable** | **Recent blood glucose test** | | | **Recent glucose and consultation** | | | **Recent medical consultation** | | |
| --- | --- | --- | --- | --- | --- | --- | --- | --- | --- |
|  | **RP** | **IC 95%** | ***p*-value** | **RP** | **IC 95%** | ***p*-value** | **RP** | **IC 95%** | ***p*-value** |
| Year 2019 (ref. 2013) | 1.07 | 1.06-1.08 | <0.001 | 1.09 | 1.07-1.10 | <0.001 | 1.04 | 1.03-1.04 | <0.001 |
| Sex Female (ref. Male) | 1.16 | 1.15-1.17 | <0.001 | 1.21 | 1.20-1.23 | <0.001 | 1.13 | 1.12-1.13 | <0.001 |
| Age (years; ref. 18-24) |  |  |  |  |  |  |  |  |  |
| 25-39 | 1.07 | 1.05-1.10 | <0.001 | 1.07 | 1.05-1.10 | <0.001 | 1.00 | 0.98-1.01 | 0.717 |
| 40-59 | 1.17 | 1.14-1.20 | <0.001 | 1.17 | 1.14-1.20 | <0.001 | 0.99 | 0.98-1.01 | 0.306 |
| ≥ 60 | 1.25 | 1.22-1.28 | <0.001 | 1.25 | 1.22-1.29 | <0.001 | 1.02 | 1.00-1.03 | 0.014 |
| Race/color (ref. White) |  |  |  |  |  |  |  |  |  |
| Black | 0.97 | 0.95-0.99 | 0.001 | 0.97 | 0.95-0.99 | 0.004 | 0.99 | 0.98-1.00 | 0.133 |
| Mixed-race | 0.97 | 0.96-0.98 | <0.001 | 0.97 | 0.95-0.98 | <0.001 | 0.99 | 0.98-1.00 | 0.031 |
| Yellow | 0.95 | 0.90-1.00 | 0.055 | 0.93 | 0.88-0.99 | 0.023 | 0.97 | 0.94-1.00 | 0.028 |
| Indigenous | 0.99 | 0.92-1.07 | 0.842 | 1.01 | 0.93-1.10 | 0.771 | 1.03 | 0.99-1.07 | 0.144 |
| Education (ref. Incomplete elementary) |  |  |  |  |  |  |  |  |  |
| Complete elementary | 1.04 | 1.02-1.06 | <0.001 | 1.03 | 1.01-1.05 | 0.009 | 1.02 | 1.01-1.04 | <0.001 |
| Complete high school | 1.13 | 1.11-1.14 | <0.001 | 1.12 | 1.10-1.14 | <0.001 | 1.04 | 1.03-1.05 | <0.001 |
| Complete higher education | 1.17 | 1.15-1.18 | <0.001 | 1.16 | 1.14-1.18 | <0.001 | 1.05 | 1.04-1.06 | <0.001 |
| Region (ref. Center-West) |  |  |  |  |  |  |  |  |  |
| Northeast | 0.98 | 0.96-0.99 | 0.012 | 0.97 | 0.95-0.99 | 0.008 | 0.99 | 0.97-1.00 | 0.025 |
| North | 0.97 | 0.95-0.99 | 0.012 | 0.95 | 0.93-0.98 | <0.001 | 0.96 | 0.94-0.97 | <0.001 |
| Southeast | 1.02 | 1.00-1.04 | 0.053 | 1.03 | 1.01-1.05 | 0.001 | 1.02 | 1.01-1.03 | <0.001 |
| South | 0.99 | 0.97-1.01 | 0.361 | 1.00 | 0.98-1.02 | 0.990 | 1.02 | 1.00-1.03 | 0.026 |
| Resident in rural area (ref. urban area) | 0.89 | 0.87-0.90 | <0.001 | 0.89 | 0.87-0.91 | <0.001 | 0.96 | 0.95-0.97 | <0.001 |
| Private health insurance No (ref. Yes) | 0.85 | 0.84-0.86 | <0.001 | 0.81 | 0.80-0.83 | <0.001 | 0.91 | 0.90-0.92 | <0.001 |
| Body mass index (ref. Low weight/Normal) |  |  |  |  |  |  |  |  |  |
| Overweight | 1.04 | 1.03-1.06 | <0.001 | 1.04 | 1.03-1.06 | <0.001 | 1.02 | 1.01-1.02 | <0.001 |
| Obesity | 1.06 | 1.05-1.08 | <0.001 | 1.06 | 1.05-1.08 | <0.001 | 1.01 | 1.01-1.02 | 0.001 |
| Hypertension Yes (ref. No) | 1.12 | 1.11-1.13 | <0.001 | 1.17 | 1.16-1.19 | <0.001 | 1.11 | 1.11-1.12 | <0.001 |

*through Poisson regression with robust variance for gender, age, education, race/color, geographic macro-region, living in an urban or rural area, having private health insurance, and the year of the survey, as well as BMI and hypertension.
